# Cavity-Free Ultrastrong Light-Matter Coupling

**DOI:** 10.1021/acs.jpclett.1c01695

**Published:** 2021-07-19

**Authors:** Philip A. Thomas, Kishan S. Menghrajani, William L. Barnes

**Affiliations:** Department of Physics and Astronomy, University of Exeter, Exeter, EX4 4QL, United Kingdom

## Abstract

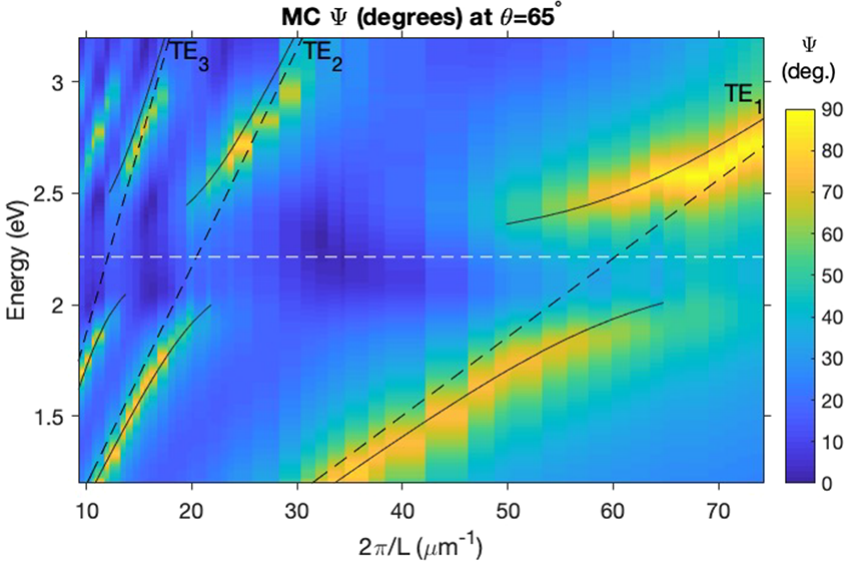

Strong coupling between light and matter can occur when the interaction strength between a confined electromagnetic field and a molecular resonance exceeds the losses to the environment, leading to the formation of hybrid light–matter states known as polaritons. Ultrastrong coupling occurs when the coupling strength becomes comparable to the transition energy of the system. It is widely assumed that the confined electromagnetic fields necessary for strong coupling to organic molecules can only be achieved with external structures such as Fabry–Pérot resonators, plasmonic nanostructures, or dielectric resonators. Here we show experimentally that such structures are unnecessary and that a simple dielectric film of dye molecules supports sufficiently modified vacuum electromagnetic fields to enable room-temperature ultrastrong light-matter coupling. Our results may be of use in the design of experiments to probe polaritonic chemistry and suggest that polaritonic states are perhaps easier to realize than previously thought.

Light–matter interactions can occur when an ensemble of molecular resonators is placed in a confined electromagnetic field. If the molecular resonance and confined electromagnetic field mode occur at similar energies, they can become coupled. When the coupling strength exceeds the losses of the uncoupled modes, the system can enter the so-called strong coupling regime.^1−3^ In this regime, two hybrid states called the upper and lower polariton bands appear, separated by the Rabi splitting energy Ω, inheriting both molecular and photonic properties. When the coupling strength is comparable to the transition energy of the system *E*_0_ (typically taken to be when Ω/*E*_0_ > 20%^4^) the system is said to be in the ultrastrong coupling regime. Recent studies have explored the effect of strong and ultrastrong coupling on phenomena such as lasing,^5^ Bose–Einstein condensation,^6^ and the modification of the emission^7,8^ and absorption^9^ of light in organic optoelectronic devices, while the nascent field of polaritonic chemistry explores the possibility of the coupling of molecular energy levels and light in modifying chemical processes.^10−14^ Understanding the effect of strong coupling on exciton transport in organic systems could also lead to new insights in photosynthesis.^15,16^

Strong coupling of light and organic molecules has been extensively studied, but experiments have typically relied on external structures such as planar microcavities,^1,7,9,10,16−18^ plasmonic nanostructures,^2,8,19,20^ and dielectric resonators (including Mie resonators^21^ and dielectric slabs^22^). The use of these structures has provided valuable insights into strong light–matter coupling but they are generally impractical for applications such as polaritonic chemistry, where they restrict access to the molecules involved. Furthermore, the high level of precision required when designing these structures (such as the thickness of a microcavity or the geometric parameters of a plasmonic lattice) implies that polaritonic effects are unlikely to play a significant role in natural processes.

In this work, we show that strong light–matter coupling can occur in extraordinarily simple systems. We experimentally demonstrate ultrastrong coupling between the molecular resonances and confined electromagnetic fields in thin (<1 μm) soft dielectric films with visible light at room temperature. Our results rely on leaky modes arising from the impedance mismatch between the dielectric thin film and a silicon substrate. Georgiou et al.^22^ have previously explored the use of leaky modes in strong coupling with an external dielectric slab microcavity. Similar strong coupling has been observed in transition metal dichalcogenide (TMDC) films^23,24^ (where Fabry–Pérot resonances within the film can couple to the excitonic resonances in the TMDC), but these results are difficult to carry over to polaritonic chemistry. Unlike the recent study of strong coupling in self-assembled organic semiconductors by Rao et al.,^25^ our sample fabrication only uses spin-coating, and our coupling strengths are an order of magnitude greater. Our results suggest that polaritonic effects might be much more prevalent than previously thought,^26^ paving the way for simplified strong coupling experiments.

We studied dielectric thin films of organic molecules deposited on silicon substrates by spin-coating (schematic in Figure 1a; see Supplementary Section S1 for fabrication details). We used spiropyran (SPI), a widely used molecule in strong coupling experiments.^10,27−29^ SPI is a transparent molecule that, when exposed to ultraviolet radiation, undergoes photoisomerisation to merocyanine (MC), which has a strong, broad absorption peak at 2.22 eV (molecular structure and transmission spectrum in Supplementary Section S2). MC can then be reconverted to SPI after exposure to visible radiation.^30^ The optical constants for our SPI and MC films are given in Figure 1c; they were determined using spectroscopic ellipsometry (with SPI fitted to a Cauchy dielectric model and MC to a Lorentzian model - see Supplementary Section S1 for fit parameters). The measured optical constants suggest that MC can support bulk polaritons^26^ with a Rabi splitting large enough to allow ultrastrong coupling (see Supplementary Section S3). The large contrast in permittivity between the SPI/MC films (ϵ_SPI_ = 2.58 at 2.22 eV) and the Si substrate (ϵ_Si_ = 16.3 at 2.22 eV), and between the SPI/MC films and the air superstrate leads to the formation of a series of leaky modes in the film for both transverse electric (TE) and transverse magnetic (TM) polarized light. Our “leaky modes” are leaky in the sense that, while tied to the layer of interest, they are radiative in the upper half space. Our nomenclature of “leaky modes” is common in the field,^31^ although we note that these modes are also referred to as quasi-normal modes.^32^

**Figure 1:**
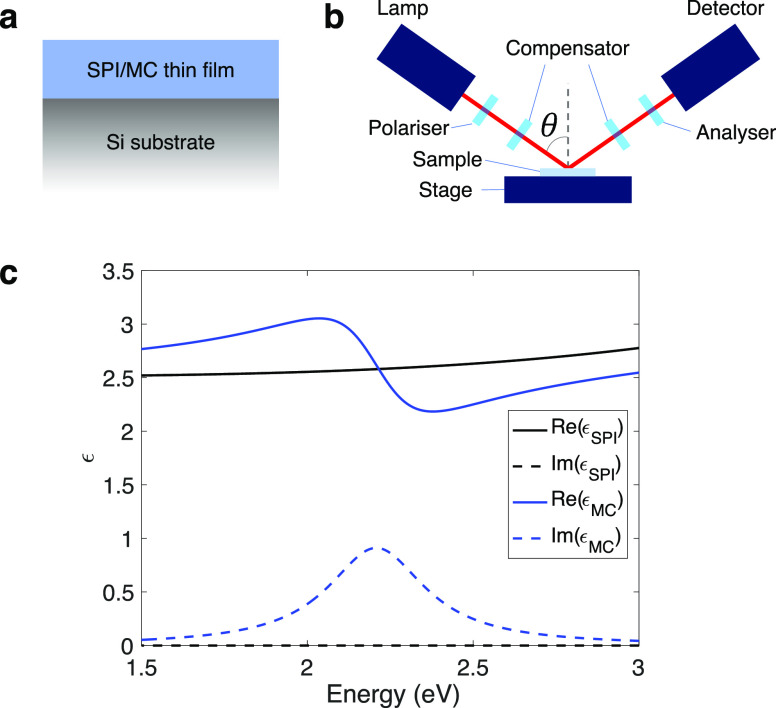
(a) Sample design showing a thin film of SPI/MC on a silicon substrate. (b) Schematic of ellipsometer arrangement for the optical measurements. (c) Real (solid lines) and imaginary (dashed lines) of the permittivities of SPI (black) and MC (blue), as derived from ellipsometry for a film of thickness 109 nm.

We characterized our samples using spectroscopic ellipsometry (schematic in Figure 1b). Ellipsometry measures the complex reflection ratio ρ in terms of the parameters Ψ and Δ:^33^
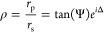
1*r*_p_ and *r*_s_ are the Fresnel reflection coefficients for p- and s-polarized light, respectively. tan(Ψ) is the amplitude of ρ and gives the ratio of the moduli of *r*_p_ and *r*_s_; Δ is the difference in the phase shifts undergone by p- and s-polarized light upon reflection. Ellipsometry is an ideal tool for our experiment.^28^ It allows one to clearly distinguish between TM leaky modes (when *r*_p_ → 0, tan(Ψ) → 0 and so Ψ → 0°) and TE leaky modes (when *r*_s_ → 0, tan(Ψ) → ∞ and so Ψ → 90°) in the same amplitude spectrum. Each ellipsometry spectrum can be used to both characterize any strong coupling and determine the thickness and optical constants of the sample under interrogation. Our choice of ellipsometer (J. A. Woollam Co. M-2000X) allows us to collect spectra over a range of steeper incident angles (45°–75°) at which leaky modes occur; its Xe arc lamp emits a small quantity of ultraviolet radiation which allows us to easily measure the transition from SPI to MC (and so the transition from weak to strong coupling). Additionally, since ellipsometry measures the ratio of two quantities it is a very low-noise measurement technique.

In Figure 2 we plot Ψ spectra for (a) SPI and (b) MC films for film thicknesses in the range 84 nm < *L* < 680 nm and for a fixed angle of incidence θ = 65°. Both plots are reproduced well by calculations (see Figures 2c,d). These calculations are based on a Fresnel approach, similar to the commonly used transfer-matrix approach. The corresponding plots of ellipsometric phase data are given in Supplementary Section S4. Plotting data for such a large range of thicknesses allows us to observe the coupling of the first-, second-, and third-order TE modes to the MC resonance. Since the reflection of p-polarized light at a SPI/MC-Si interface is considerably weaker than for s-polarized light,^34^ the TM leaky modes have a lower quality factor than the equivalent-order TE modes. We therefore restrict our analysis to the TE modes, which all show a clear anticrossing, a key signature of strong coupling.^2^ The lack of evidence for any mid-polariton bands in Figure 2 suggests that the polariton branches in our system are best modeled by a 2*N* coupling matrix.^35^ In this case, the coupling between the MC resonance and the *j*th-order TE leaky mode can be described by the following matrix equation:
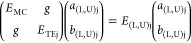
2where *E*_MC_ is the energy of the MC resonance, *g* ∼ Ω/2 is the coupling strength, *E*_TE*j*_ is the *j*th-order TE leaky mode energy, *E*_(L,U)*j*_ are the energies of the lower and upper polariton bands associated with the *j*th TE leaky mode, and |*a*_(L,U)*j*_|^2^ and |*b*_(L,U)*j*_|^2^ are the Hopfield coefficients describing the mixing of the MC resonance with the *j*th TE leaky mode.

**Figure 2:**
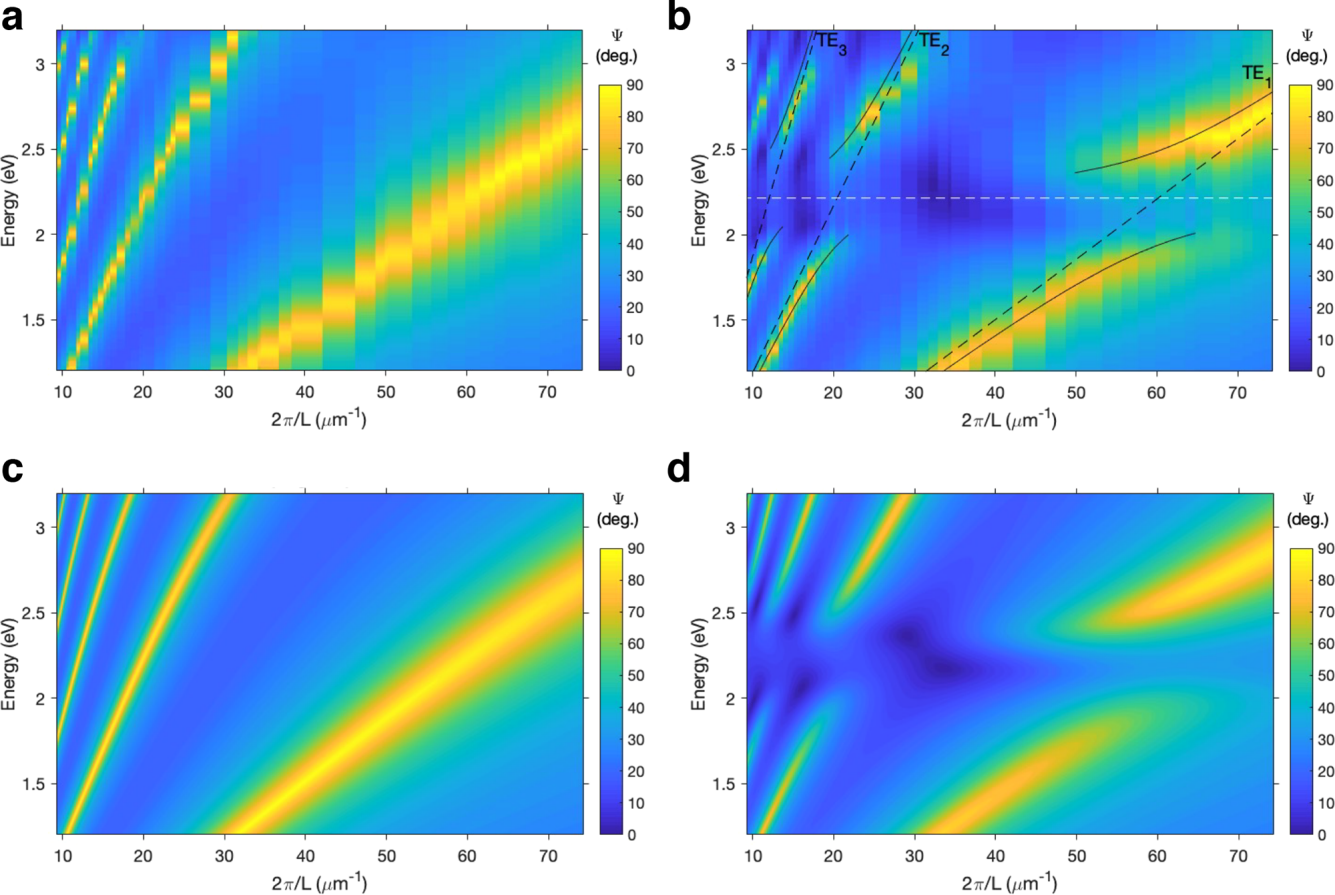
Experimental (a,b) and calculated (Fresnel approach) (c,d) dispersion plots constructed using the ellipsometric parameter Ψ for (a,c) SPI and (b,d) MC films over a range of thicknesses *L* at fixed angle θ = 65°. The lines in (b) indicate the positions of the uncoupled MC resonance (dashed white line), TE leaky modes (dashed black lines) and upper and lower polariton bands (solid black lines) calculated using the coupled oscillator model (Ω = 550 meV).

The fits are plotted as the black lines in Figure 2b. The best fit to the data was obtained with Ω = 550 meV. This fulfils the strong coupling resolution criterion:^2^
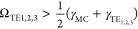
3where γ_MC_ ≈ 360 meV is the line width of the MC resonance and γ_TE_1__ ≈ 310 meV, γ_TE_2__ ≈100 meV and γ_TE_3__ ≈ 60 meV are the line widths of TE_1,2,3_ leaky modes, respectively. This value of Ω also fulfils the ultrastrong coupling criterion:^4^
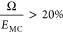
4where *E*_MC_ is the MC molecular resonance. Analyzing the data for a fixed, oblique incident angle produces an overestimate of Ω which must be taken into account in ultrastrong coupling experiments. Supplementary Section S5 shows a dispersion plot (energy versus in-plane wavevector) of the TE_2_ leaky mode coupling to MC. In this case, the best fit is achieved with Ω = 500 meV, which still fulfils the ultrastrong coupling criterion. In Supplementary Section S6, we plot ρ to study the transition from weak to strong coupling of the MC resonance and TE_2_ leaky mode and observe a change in ellipsometric topology consistent with our previous observations in strong coupling experiments.^28^ In Supplementary Section S7, we model the absorption of MC and show that the absorption of MC is modified in the Si/MC/air geometry, suggesting that the observed anticrossing is a signature of genuine strong coupling and not just an artifact of ellipsometry.

Leaky electromagnetic modes are, by definition, not an obvious way of confining electromagnetic fields for strong coupling experiments, yet we have shown that they allow for coupling in the strong and ultrastrong coupling regimes. Calculations suggest the impedance mismatch between the SPI/MC film and the Si substrate is high enough to generate a strong electromagnetic field within the film. We have compared the electric field enhancement from a TE_2_ mode in SPI with the field enhancement arising from a second-order microcavity mode in a metal-clad microcavity. Figure 3a compares the peak electromagnetic field enhancement of the TE_2_ leaky mode in our system with that of the second-order microcavity mode in a metal-clad cavity for 0° ≤ θ ≤ 85°, calculated using finite-element modeling (COMSOL Multiphysics 5.5). In both cases, the substrate was silicon and the SPI thickness was 294 nm (to match the TE_2_ mode in Supplementary Sections S5–S6). In the microcavity calculations, the SPI layer was placed between two Ag films of thickness 40 nm. At normal incidence, the peak field enhancements of the microcavity and leaky modes differ by less than a factor of 2. As the incident angle is increased, the field enhancement increases for leaky modes and decreases for microcavity modes. For θ = 65° (the angle of incidence used for all our experiments), the leaky mode field enhancement in our system is as much as 77% of the cavity mode field enhancement in a metal-clad microcavity. This is roughly consistent with the difference between the Rabi splitting measured for coupling between the TE_2_ and MC resonance in Supplementary Section S5 (Ω = 500 meV) and the Rabi splitting previously measured in a metal-clad microcavity by Schwartz et al.^27^ (Ω = 700 meV). We note that the impedance mismatch between Si and SPI/MC is not dissimilar to the impedance mismatch between TMDCs and the SiO_2_ substrate used to achieve self-coupling of light and matter in TMDCs.^24^ An important difference between the two systems is that while self-coupling in TMDCs relies on their high (15–20) dielectric constants to confine light, SPI/MC films have a relatively low dielectric constant (2.5). Calculations presented in Supplementary Section S8 suggest that, provided the substrate permittivity is greater than the thin film’s permittivity, only a modest impedance mismatch between substrate and dielectric film is necessary to achieve strong coupling in a substrate/dielectric/air structure. However, repeating the SPI-MC experiment in Figure 2, but this time using a glass substrate (in which case the dielectric film has a slightly greater permittivity than the substrate), produces no clear signature of strong coupling (see Supplementary Section S9); in this type of structure, fully guided modes and self-hybridization are possible beyond the light-line.^26,36^

**Figure 3:**
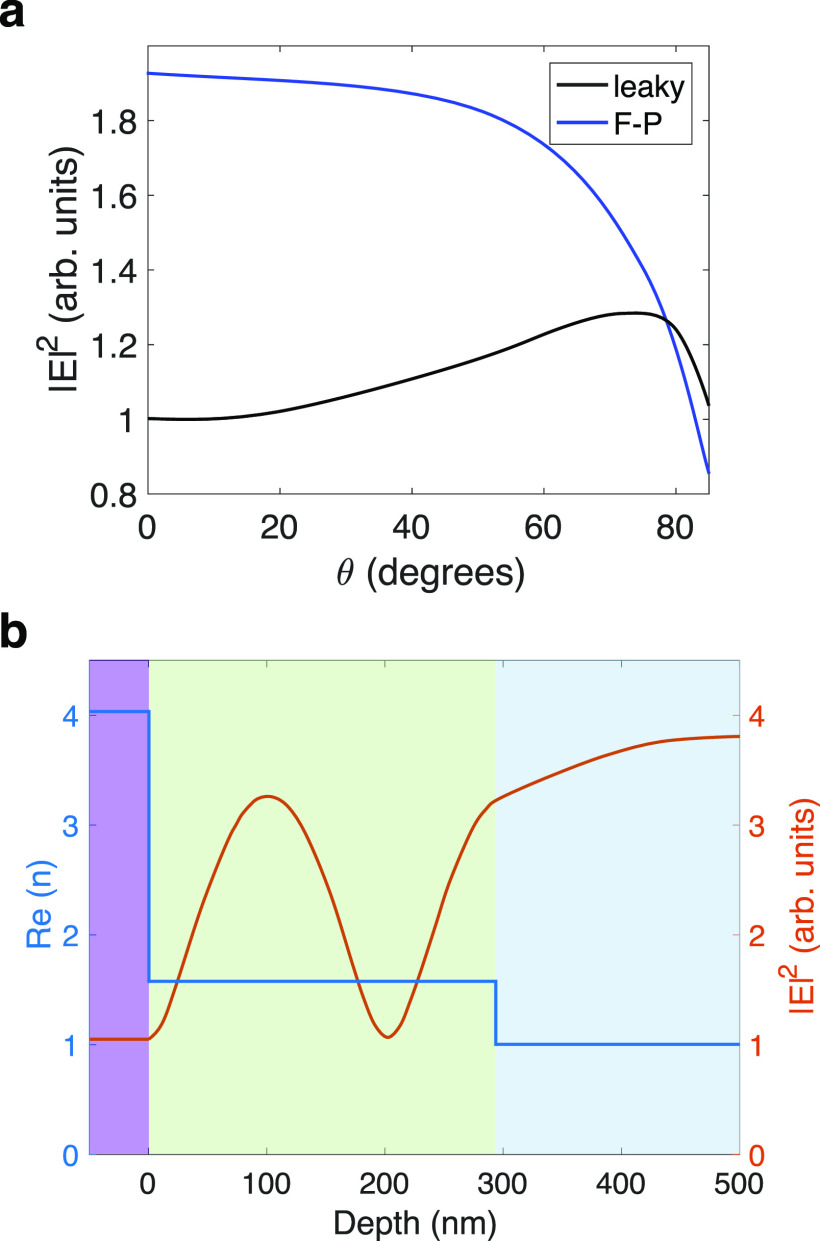
(a) Maximum electric field enhancement as a function of incident angle associated with the second-order Fabry–Pérot cavity resonance in a Si substrate/Ag(40 nm)/SPI(294 nm)/Ag(40 nm)/air structure (blue line) and the second-order TE leaky mode in a Si substrate/SPI(294 nm)/air structure (black line). (b) Electric field profile associated with the second-order TE leaky mode in a Si substrate/SPI (294 nm)/air structure for light incident at θ = 65°. The different colored regions indicate each layer of the structure (Si, purple; SPI, green; air, blue).

A further difference between cavity modes and leaky modes is their electromagnetic field profiles. At the ends of a metal-clad microcavity the electromagnetic field profile tend to zero (see Supplementary Section S10). In contrast, the field profile of a leaky mode (see Figure 3b for the profile of a TE_2_ mode) remains approximately constant across the dielectric/air interface and extends outside the film, suggesting that molecules placed on top of the dielectric film could interact with the same leaky mode as molecules inside the film.^22^

The implications of these results are far-reaching, and they suggest that the properties of dye molecules in a thin film can be modified by varying the film’s thickness. Our work suggests a cavity-free platform in which the effect of strong coupling on reaction rates might more easily be explored. Our results also suggest that, provided a sufficiently strong impedance mismatch is present, the more complex Fabry–Pérot cavities, self-assembled, and plasmonic nanostructures frequently used in strong coupling experiments might be complimented by the simple slab geometry shown here. Furthermore, our results suggest care must be taken when designing control samples used to determine the influence of strong coupling on chemical processes: it is possible, for example, that polaritonic modes will remain even after removing the top mirror from a metal-clad microcavity.^9,10,17^ In summary, our results suggest a new, simple way of achieving strong and ultrastrong light–matter coupling which will be useful in polaritonic chemistry experiments.

## Supporting Information Available

Experimental methods; SPI/MC chemical structure and MC transmittance spectrum; calculation of bulk polariton splitting in MC; SPI/MC on Si cos(Δ) plot; SPI/MC on Si ellipsometry spectra plotted as a function of in-planewavevector; SPI/MC on Si change in ellipsometric topology; modeling of absorption of SPI/MC film on Si; effect of substrate permittivity on strong coupling; SPI/MC on SiO_2_ Ψ and Δ plots; field profiles of Fabry–Perot and leaky modes (PDF)
