# Supplementary material for: Cavity-Free Ultrastrong Light-Matter Coupling
Source: J Phys Chem Lett. 2021 Jul 19;12(29):6914–8. doi: 10.1021/acs.jpclett.1c01695 (PMC8327311; doi:10.1021/acs.jpclett.1c01695)
Supplement: Supplementary file 1 — jz1c01695_si_001.pdf [file jz1c01695_si_001.pdf]

Supplementary Information for:

Cavity-Free Ultrastrong Light-Matter Coupling

Philip A. Thomas, Kishan S. Menghrajani and William L. Barnes

Department of Physics and Astronomy, University of Exeter,
Exeter, EX4 4QL, United Kingdom

Contents

S1. Methods **2**

S2. SPI/MC chemical structure and MC transmittance spectrum **3**

S3. Calculation of bulk polariton splitting in MC **4**

S4. SPI/MC on Si $\cos(\Delta)$ plot **5**

S5. SPI/MC on Si ellipsometry spectra plotted as a function of in-plane wavevector **6**

S6. SPI/MC on Si change in ellipsometric topology **8**

S7. Modelling of absorption of SPI/MC film on Si **10**

S8. Effect of substrate permittivity on strong coupling **12**

S9. SPI/MC on SiO_2 Ψ and Δ plots **14**

S10. Field profiles of Fabry-Perot and leaky modes **16**

S1. Methods

SPI/MC film fabrication

Polymethyl methacrylate (PMMA, molar weight 450 000) was used as a host matrix for SPI. PMMA was dissolved in toluene. SPI was then dissolved in the PMMA-toluene solution with a weight ratio of 3:2 SPI to PMMA. SPI/PMMA films were deposited on a silicon wafer by spin-coating three layers each with a spin speed of 2000 rpm. This produced film thicknesses over the range 84–681 nm.

Spectroscopic ellipsometry

Spectroscopic ellipsometry was carried out using a J. A. Woollam Co. M-2000XI ellipsometer with which we measured the ellipsometric parameters Ψ and Δ in the wavelength range 210–1690 nm, with a wavelength step of 1.5 nm for 210–1000 nm and 3.5 nm for 1000–1690 nm. Ψ gives the ratio of the field reflection coefficients for p- and s-polarised light (the moduli of r_p and r_s, the complex Fresnel reflection coefficients for p- and s-polarised light respectively) and Δ is the phase difference between the same coefficients such that $r_p/r_s = \tan(\Psi)e^{i\Delta}$. Since ellipsometry measures the ratio of two signals it cancels out a lot of noise from the source, making it a very sensitive measuring technique. The light source in the M-2000XI was a 75 W Xe arc lamp which produced a a smooth ultraviolet continuum[1] that was used to convert SPI to MC.

Optical constants of SPI and MC

Optical constants of SPI and MC were determined using CompleteEASE®[2]. The permittivity of SPI (ϵ_{SPI}) was modelled as a Cauchy dielectric:

$$\epsilon_{\mathrm{SPI}} = \left(A + B\omega^2 + C\omega^4\right)^2$$

where $A = 1.584$, $B = -4.171 \times 10^{-34}$ rad^{-2} s^2 and $C = 2.105 \times 10^{-64}$ rad^{-4} s^4. The permittivity of MC (ϵ_{MC}) was modelled with a Lorentz oscillator (ϵ_L) and a pole in the ultraviolet (ϵ_{UV}):

$$\begin{aligned}\epsilon_{\mathrm{MC}} &= \epsilon_\infty + \epsilon_{\mathrm{UV}} + \epsilon_L \\ &= \epsilon_\infty + \frac{A_{\mathrm{UV}}}{E_{\mathrm{UV}}^2 - (\hbar\omega)^2} + \frac{f}{E_L^2 - (\hbar\omega)^2 - i\hbar\omega\gamma_L},\end{aligned}$$

where $\epsilon_\infty = 0.839$, $A_{\mathrm{UV}} = 102.133$ eV^2, $E_{\mathrm{UV}} = 7.970$ eV, $f = 0.7226$ eV^2, $E_L = 2.215$ eV, $\gamma_L = 0.3587$ eV.

S2. SPI/MC chemical structure and MC transmittance spectrum

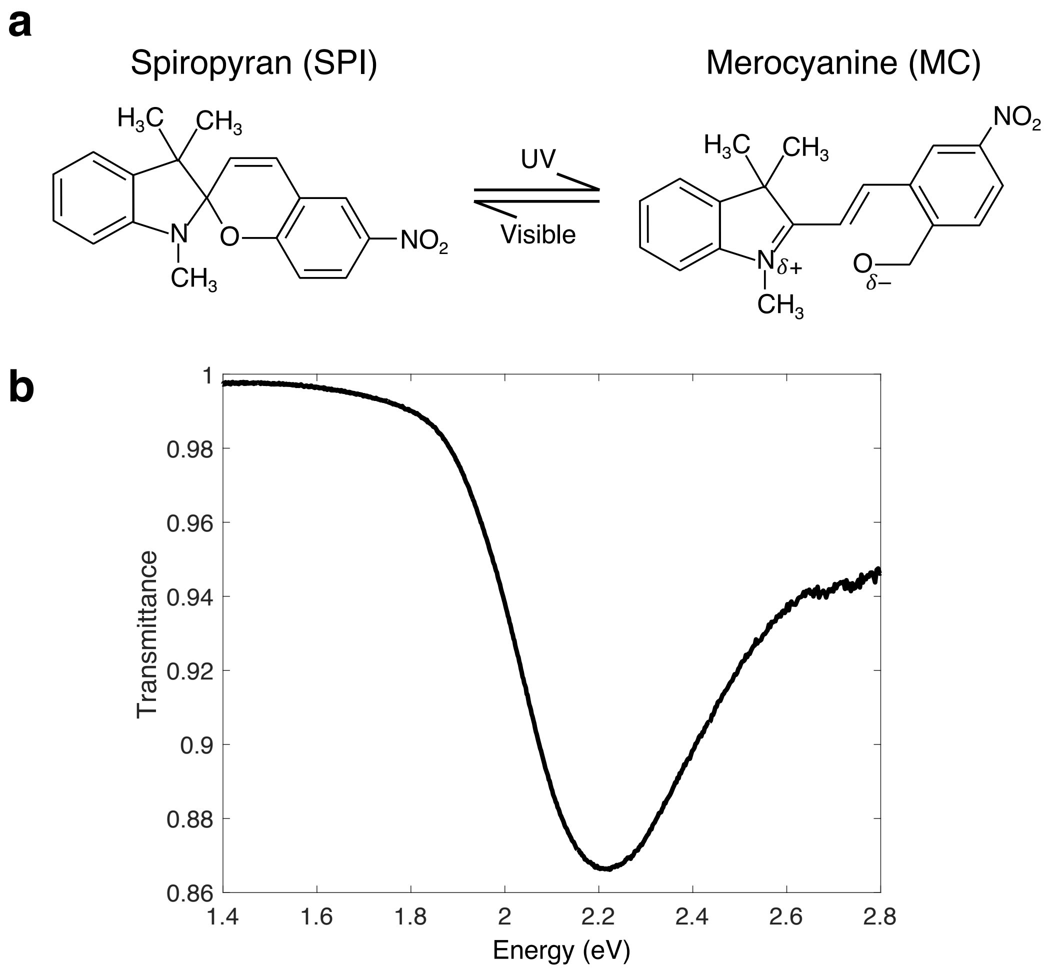

Figure S1: (a) Chemical structures of spiropyran and merocyanine. (b) Transmittance through a merocyanine (MC) film (thickness 150 nm) spin-coated on a glass substrate, normalised against transmission for an uncoated substrate (see Methods for fabrication).

S3. Calculation of bulk polariton splitting in MC

By following the work of Canales *et al.*[3], we show that MC supports bulk polaritons with a coupling strength that fulfils both the strong and ultrastrong criteria. Using the same notation as for the optical constants above, bulk polaritons appear when the following equation is satisfied:

$$\frac{\sqrt{f}}{2\sqrt{\epsilon_{\mathrm{BG}}}} = g_0 > \frac{\gamma_L}{4},$$

where $\epsilon_{\mathrm{BG}} = \epsilon_\infty + \epsilon_{\mathrm{UV}}(E_L)$ is the background (non-Lorentzian) contribution to the permittivity at the MC resonance energy and g_0 is the bulk coupling strength at zero detuning. Using the above optical constants for MC we calculate $g_0 = 265$ meV which easily fulfils the above criterion for bulk polaritons and the criteria for strong and ultrastrong coupling. This value of g_0 agrees very well with the coupling strength derived from a coupled oscillator fit to the dispersion plots in Supplementary Figure S3 ($\Omega/2 = 250$ meV).

S4. SPI/MC on Si $\cos(\Delta)$ plot

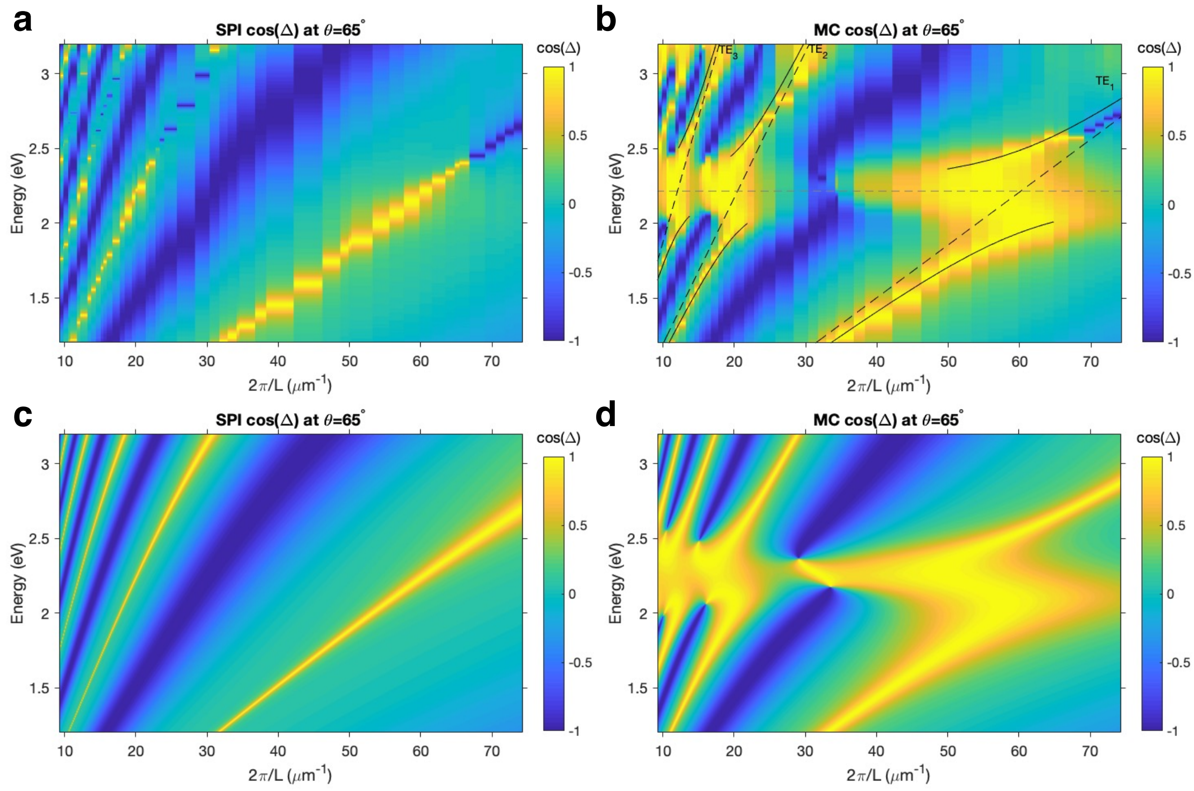

Figure S2: Experimental (a,b) and calculated (c,d) dispersion plots constructed using the ellipsometric parameter $\cos(\Delta)$ for (a,c) SPI and (b,d) MC films over a range of thicknesses L at fixed angle $\theta = 65°$. The colour-plot data were calculated using a Fresnel-approach, as discussed in the main text. The lines in (b) indicate the positions of the uncoupled MC resonance (dashed grey line), TE leaky modes (dashed black lines) and upper and lower polariton bands (solid black lines) calculated using the coupled oscillator model ($\Omega = 550$ meV).

The amplitude plot in Main Figure 2b shows two distinct polariton branches separated by a stop-gap for each split mode. However, the phase data here reveals back-bending within the stop-gap region connecting the split modes. Back-bending was reported in early strong coupling experiments[4, 5], but it was later determined that these measurements were an artefact of the measurement technique, caused by the finite widths of the split modes[6]. The dispersion relationship for coupled lossy modes predicts a back-bending feature connecting the upper and lower polariton bands[7]; this back-banding is highly suppressed, making it difficult to resolve in experimental intensity data. Since even a low-amplitude feature will have an associated phase jump, it is not surprising that back-banding can only be observed in our phase data.

S5. SPI/MC on Si ellipsometry spectra plotted as a function of in-plane wavevector

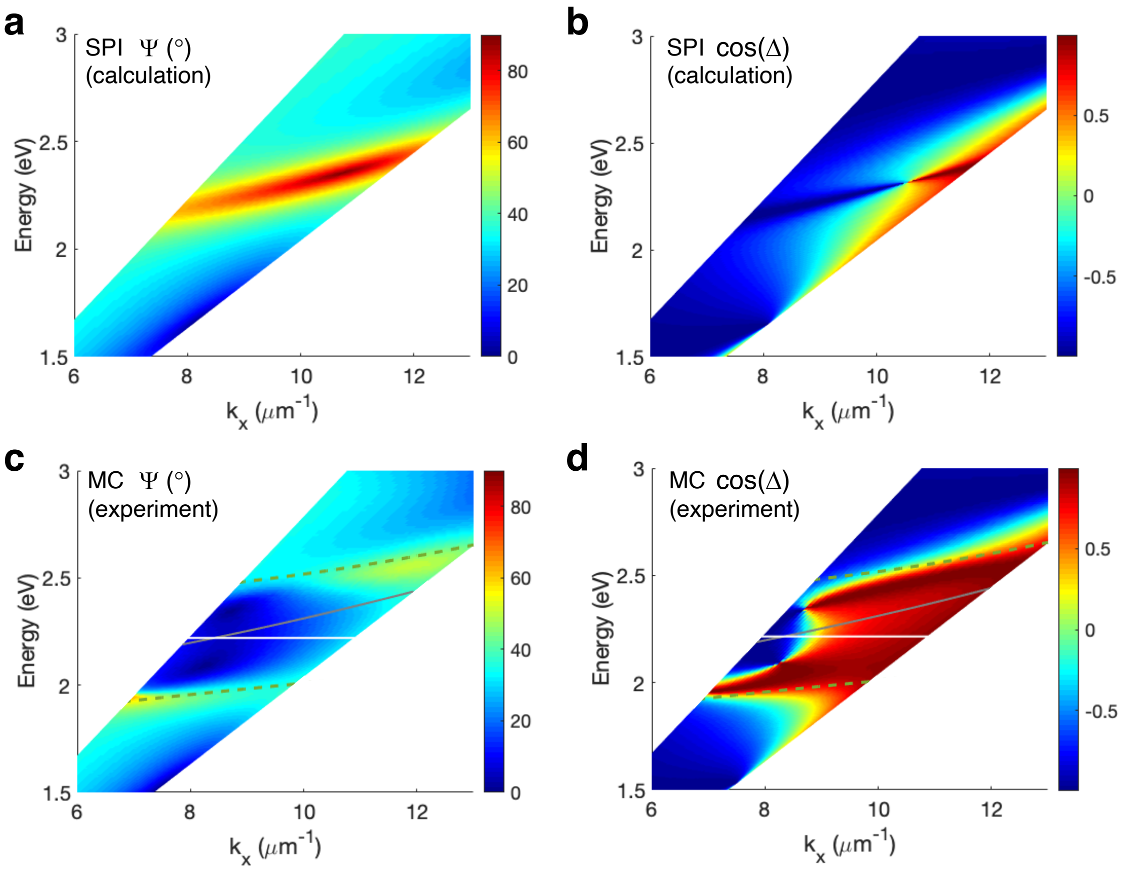

Figure S3: Dispersion plots (energy versus $k_x = \frac{2\pi}{\lambda}\sin(\theta)$ where λ = wavelength) of Ψ and $\cos(\Delta)$ for SPI and MC films (both of thickness 294 nm).

To determine the coupling strength of our MC-leaky mode system at zero detuning, we construct dispersion plots (energy versus $k_x = \frac{2\pi}{\lambda}\sin(\theta)$ where λ = wavelength) of Ψ and $\cos(\Delta)$ for SPI and MC films (both of thickness 294 nm). The plots were constructed from a set of ellipsometry spectra over the range $45° \leq \theta \leq 75°$. The SPI data in figures (a) and (b) were generated with CompleteEASE®[2] using the experimentally-derived optical constants in Main Figure 1c. (It is not possible to build a SPI dispersion plot using our ellipsometer since significant quantities of SPI turn into MC after just a few measurements.) At $(E,\, k_x) = (10.70\ \mu\text{m}^{-1},\, 2.35\ \text{eV})$ $(\theta = 63.5°)$, $\Psi = 89.60°$, indicating that around this point r_s is close to if not exactly 0. At this point in figure (b) we observe the kind of phase singularity associated with points of zero amplitude, suggesting that the leaky mode is better confined at the air-SPI interface that one might expect.

The MC Ψ and $\cos(\Delta)$ plots in figures (c)-(d) show a clear anti-crossing. The positions of the uncoupled MC resonance and TE_2 leaky mode are indicated by the solid white and grey lines, respectively. The dashed green lines are fits to the coupled oscillator model (see Methods). We achieve the best fit with a Rabi splitting of $\Omega = 500$ meV which fulfils

both

$$\Omega > \frac{1}{2}\left(\gamma_{\mathrm{MC}} + \gamma_{\mathrm{TE2}}\right), \tag{1}$$

the commonly-used strong coupling criterion[7], and,

$$\frac{\Omega}{E_{\mathrm{MC}}} > 20\%, \tag{2}$$

the commonly-used ultrastrong coupling criterion[8].

The calculated positions of the upper and lower polariton bands match well with the phase jumps in figure (d). Additionally, we note the presence of two new phase singularities at lower k_x values between the polariton branches. (c) reveals that these correspond to two points ((E, k_x) = (8.72 μm^{-1}, 2.35 eV) and (8.23 μm^{-1}, 2.09 eV); $\theta = 47.0°$ and $51.0°$) where $\Psi \rightarrow 0°$ and so $r_p \rightarrow 0$. They are therefore unlikely to be directly associated with coupling to the TE_2 mode. Such strong absorption at two distinct points on the $E - k_x$ plot also rules out the effect of MC. Instead, we suggest that these points are associated with a TM leaky mode that appears in two adjacent positions on the MC dispersion plot where the permittivity of MC rapidly changes (see main paper figure 1c). The presence of the higher energy phase singularity distorts the phase response of the upper polariton in (d), which otherwise matches well with the calculated position of the upper polariton band.

S6. SPI/MC on Si change in ellipsometric topology

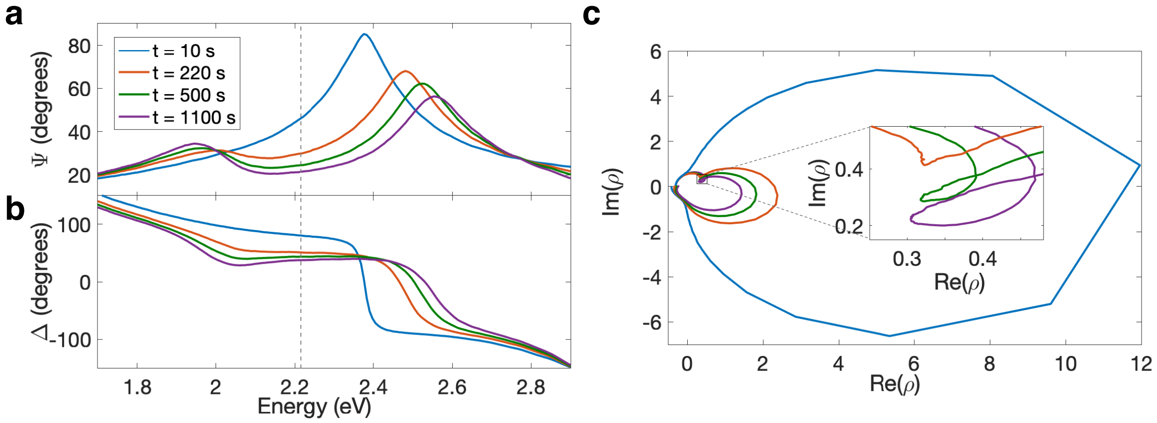

Figure S4: The ellipsometric parameters (a) Ψ, (b) $\cos(\Delta)$ and (c) ρ for a SPI/MC film (thickness 294 nm) on Si after exposure to the ellipsometer lamp's UV radiation for 10 seconds (blue line), 220 seconds (orange line), 500 seconds (green line) and 1100 seconds (purple line). All spectra are measured for light at an incident angle of $\theta = 65°$. The vertical grey dashed lines in figures (a,b) indicate the spectral position of the MC resonance. In (c) ρ has been plotted for energies from 1.5 eV to 3.0 eV.

In Supplementary Figure S4 we plot ellipsometry spectra for a 294 nm thickness SPI film after exposure to the ellipsometer's light source for 10 seconds (blue line), 220 seconds (orange line), 500 seconds (green line) and 1100 seconds (purple line). All spectra are measured for light at an incident angle of $\theta = 65°$. The vertical grey dashed lines in figures (a,b) indicate the spectral position of the MC resonance. In (c) ρ has been plotted for energies from 1.5 eV to 3.0 eV. After 10 seconds a negligible quantity of SPI molecules have been converted to MC and a peak in Ψ is visible at just less than 2.4 eV in (a). This is the second-order TE (TE_2) leaky mode; the associated sharp jump in Δ in (b) tells us that there is close to no reflection of s-polarised light at this energy. At $\theta = 65°$ the MC resonance and TE_2 leaky mode are detuned by 160 meV; this is small compared to the linewidths of the MC molecular resonance ($\gamma_{MC} = 350$ meV) and TE_2 leaky mode ($\gamma_{TE2} = 225$ meV). At later times more SPI is converted into MC and we observe two split modes in Ψ. At $t = 1100$ s the mode splitting at $\theta = 65°$ is 608 meV. This is not the same as the Rabi splitting Ω, which is defined as the splitting at the point of zero detuning between the uncoupled modes, but suggests that the system is likely in the strong coupling regime.

In Δ (figure (b)) the initial phase jump associated with the uncoupled TE_2 leaky mode is effectively pulled apart into two phase jumps associated with each split mode. The phase jumps of each split mode are not as sharp as the uncoupled TE_2 mode because the amplitude of the coupled modes is weaker. At later times a point of inflection in the phase response appears between the two split mode responses, with a maximum at around 2.35 eV. We have previously suggested that this ellipsometric phase response allows one to differentiate between spectrally adjacent, uncoupled resonances and polariton branches

arising from strong coupling[9].

In (c) we combine the amplitude and phase responses to plot the ellipsometric parameter ρ for energies in the range 1.5 eV - 3.0 eV. The initial uncoupled TE_2 mode at $t = 10$ s produces a loop in ρ and has a maximum amplitude of $\tan(85.24°) = 12.01$. This is substantially higher than the maximum amplitudes of the other datasets because the unexposed film has substantially lower absorption. The inset in figure (c) focuses on the most interesting features at later times. As the number of MC molecules in the film increases the ρ response changes from a simple loop (blue line) to a loop with a "kink" (orange line) to a loop with an additional secondary loop (green and purple lines). We have previously suggested that, since the emergence of the secondary loop represents a change in topology of ρ, it is a signature of the system is in transition from the weak coupling regime to the strong coupling regime[9]. This implies that for $\theta = 65°$ at some point between 220 s and 500 s enough SPI molecules have been converted to MC to allow the dielectric film to enter the strong coupling regime.

S7. Modelling of absorption of SPI/MC film on Si

Here we investigate the absorption response of a MC film on a silicon substrate, through use of a Fresnel approach, equivalent to the common transfer matrix technique. Our purpose is to see whether the changes in reflection/ellipsometry parameters are related to an underlying change in absorption. To explore this question we first chose to calculate the response of light incident from within a silicon superstrate on a film of MC. For clarity we also include here some data for an SPI-based system, to act as a comparison to the MC results. The parameters for the MC (and SPI) film are given above; we took the refractive index of the silicon to be 4.0. To begin this exploration we calculate the dispersion of the modes supported by this structure, we do this by plotting the absolute value of the s-polarised Fresnel transmission (amplitude) coefficient as a function of energy and incident angle[10, 11]; the results are shown in figure S5.

To continue this exploration, and to provide an easier comparison with our experimental data, we now perform calculations similar to those in figure S5, but this time the light is incident from the air side. We looked at the situation with light incident through the Si first because the modes are better defined, and it is somewhat easier to see what is going on. Once we have the experience of looking at the Si-side data, then we are better

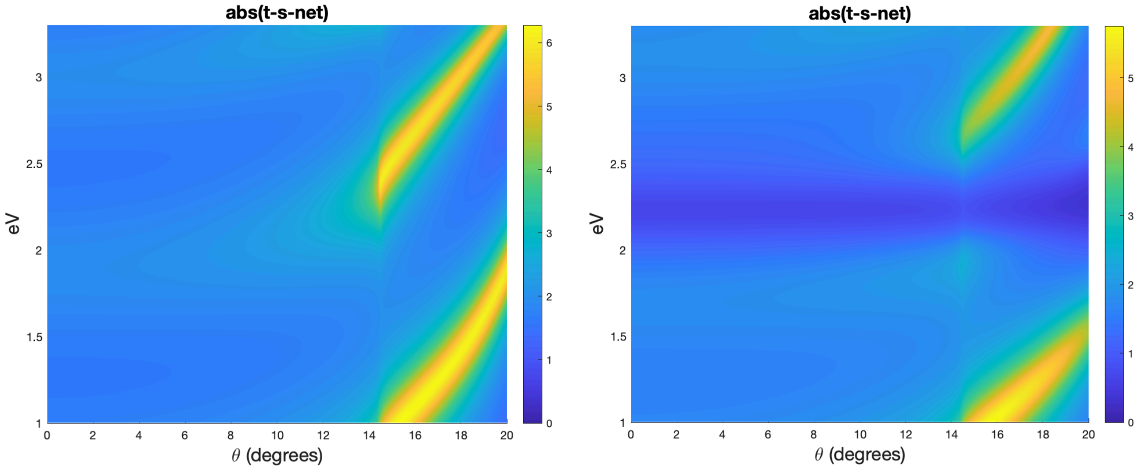

Figure S5: Fresnel transmission amplitude for s-polarised light showing the dispersion of s-polarised modes for SPI (left) and MC (right). The calculated dispersion is for a three layer system comprising: superstrate, semi-infinite silicon medium (n=4.0); 310 nm thick SPI/MC film; substrate, air (n=1.0). This is a typical prism coupling arrangement for leaky modes[12]. The colour scale shows the absolute magnitude of the (amplitude) s-polarised transmission coefficient. For this system an incident angle of 14.5 degrees corresponds to total internal reflection at a silicon/air interface. Angles lower than this correspond to leaky modes; their leaky (damped) nature is evident from their greater width. For angles greater than 14.5 degrees the modes are fully guided, and hence much narrower. In the right panel the second-order mode crosses the MC exciton energy of ∼ 2.2 eV at an angle of approx. 14 degrees, and we see a splitting of both this second-order mode.

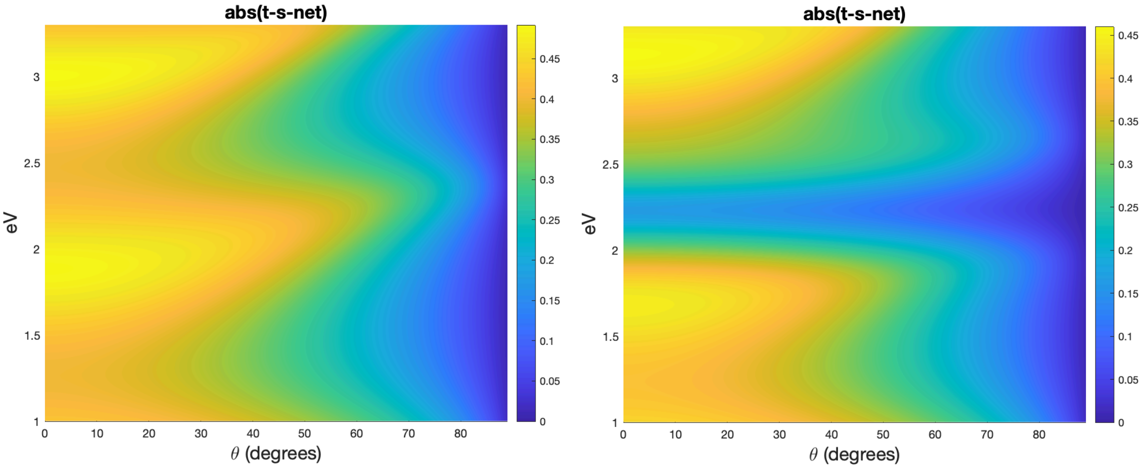

Figure S6: Fresnel transmission amplitude for s-polarised light showing the dispersion of s-polarised modes for SPI (left) and MC (right), this time calculated for light incident from the air side. The calculated dispersion is thus for a three layer system comprising: superstrate, air (n=1.0); 310 nm thick SPI/MC film; substrate, semi-infinite silicon medium (n=4.0). The colour scale shows the absolute magnitude of the (amplitude) s-polarised transmission coefficient. In the right panel the second-order leaky mode crosses the MC exciton energy of ~ 2.2 eV at an angle of approx. 65 degrees, one of the angles for which we acquired ellipsometry data.

placed to understand the air-side data, the calculated data are shown in figure S6.

We are now in a position to calculate the data we really wanted to look at here, the absorption. To do this we take a slice through the dispersion, choosing an angle of 65 degrees, where the leaky mode shows a clear splitting in the s-polarised amplitude transmission coefficient, see right-hand panel in figure S6 above. In figure S7 we plot the calculated transmittance (T) and reflectance (R) as a function of the frequency (energy) of the incident light, and infer the absorbance from the relationship $A = 1 - R - T$. Also shown in the plot below is the imaginary part of the refractive index of the MC, scaled to match the absorbance maximum. The splitting of the leaky mode is clear in the reflectance and transmittance data, but the absorption is less clear - there is still very significant absorption at around the bare exciton energies. However, comparison of the calculated absorbance with the imaginary part of the refractive index of the MC, i.e. MC's extinction coefficient, k_{MC}, shows that the absorption in the leaky system is much wider than that of bare MC, indicative of a contribution from the splitting / anti-crossing. The fact that the spectral signatures of reflection, transmission and absorption are not identical is expected when one measure the response of such systems in this way[13, 14]. To see this even more clearly we have also included another data set, this time showing the excess absorption beyind the material absorption, i.e. k_{MC}, shown by the dash-dot line.

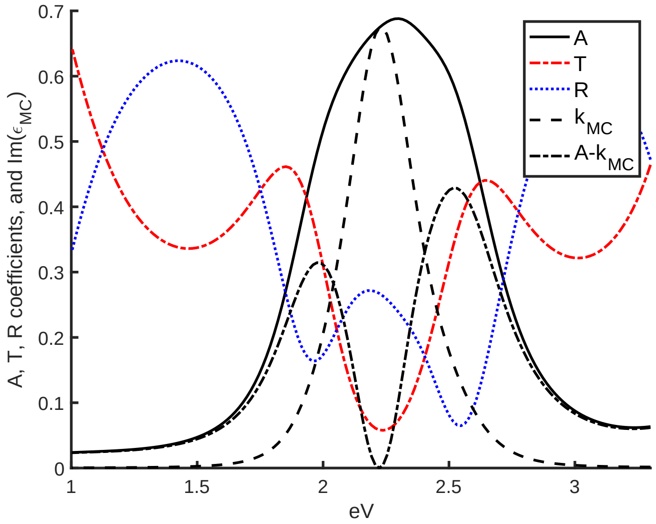

Figure S7: Transmittance (red, dash-dot), Reflectance (blue, dotted), Absorbance (black) as a function of energy for an incident angle of 65 degrees. Also shown is the imaginary part of the complex refractive index of the MC (black, dashed), i.e. the extinction coefficient. Finally, the difference between the absorption of the three layer system and the MC extinction is also plotted (black, dash-dot).

S8. Effect of substrate permittivity on strong coupling

To investigate the role of the impedance mismatch between the substrate and SPI/MC film we have used CompleteEASE®[2] to calculate Ψ and $\cos(\Delta)$ for a substrate / MC (thickness 294 nm) / air structure with variable substrate permittivity ϵ at an incident angle of 65°. The horizontal dotted line shows the position of the MC resonance; the vertical dashed line shows the real permittivity of MC at each energy. For higher values of ϵ there are clear signatures of strong coupling in both amplitude (a) and phase (b) spectra around the MC resonance energy. Although we have used a silicon substrate ($\epsilon \approx 16$) in our experiments, these calculations suggest that this structure supports strong coupling even for a much more modest ϵ (even as low as $\epsilon \approx 5$).

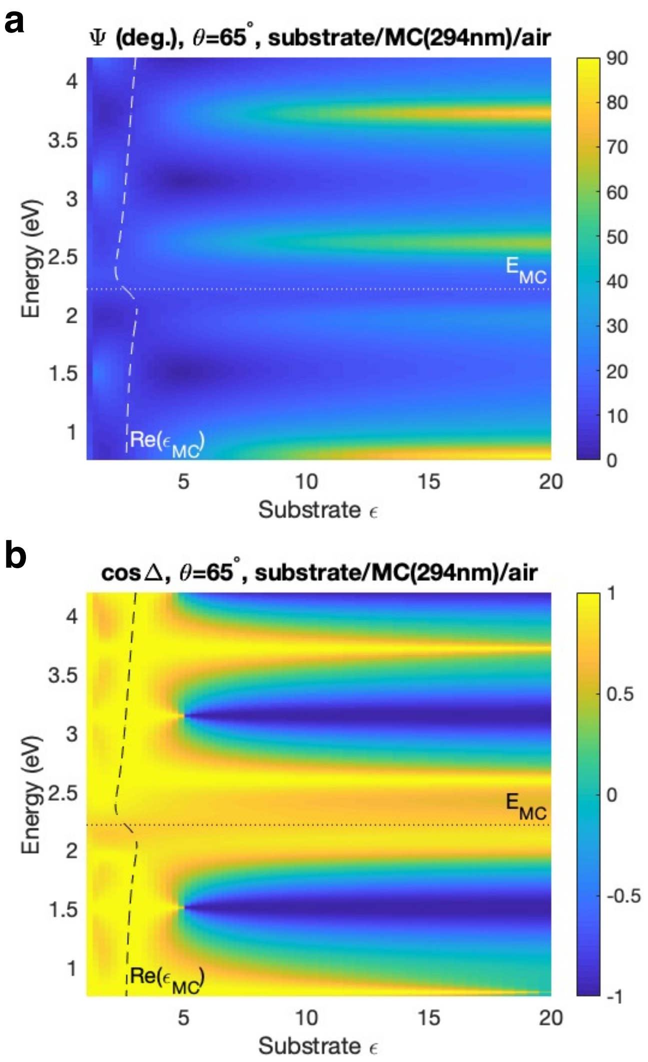

Figure S8: Calculated ellipsometric spectra for a substrate / MC (thickness 294 nm) / air structure with variable substrate permittivity ϵ at an incident angle of 65°. The horizontal dotted line shows the position of the MC resonance; the vertical dashed line shows the real permittivity of MC at each energy.

S9. SPI/MC on SiO_2 Ψ and Δ plots

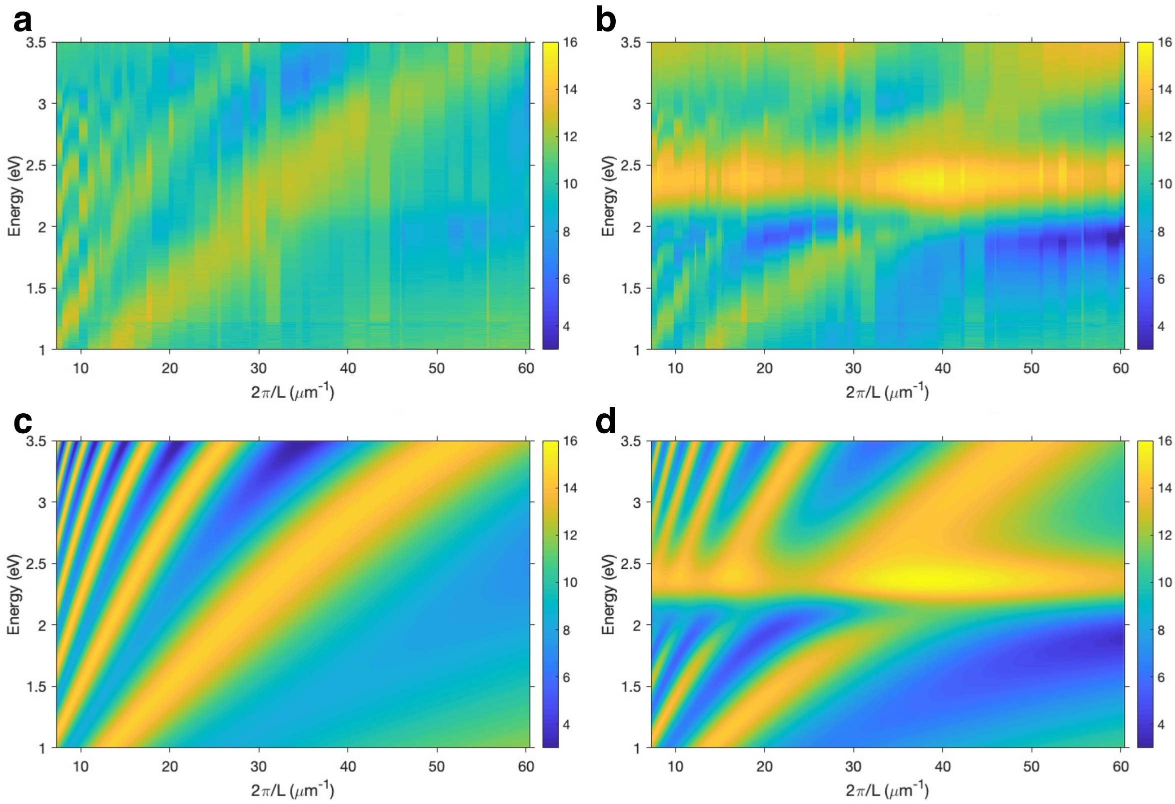

Figure S9: The ellipsometric parameter Ψ plotted for SPI (a,c) and MC (b,d) films of thickness 104 nm $< L <$ 870 nm on an amorphous glass substrate ($\epsilon = 2.3$). The corresponding phase data is plotted in Figure S10.

We repeated the SPI/MC on Si experiment using an amorphous glass (SiO_2) substrate. In Figure S9 we plot Ψ for SPI (a,c) and MC (b,d) films of thickness 104 nm $< L <$ 870 nm on an amorphous glass substrate ($\epsilon = 2.3$). The corresponding phase data is plotted in Figure S10. There is strong agreement between experiment (a-b) and modelling (c-d). The SPI film clearly shows successive TE modes. In the MC film there is no clear anticrossing around 2.2 eV. Instead, the value of Ψ for the broad MC mode increases when it intersects a TE mode. This suggests there is no strong coupling when the impedance mismatch between the SPI/MC film and the substrate is very low.

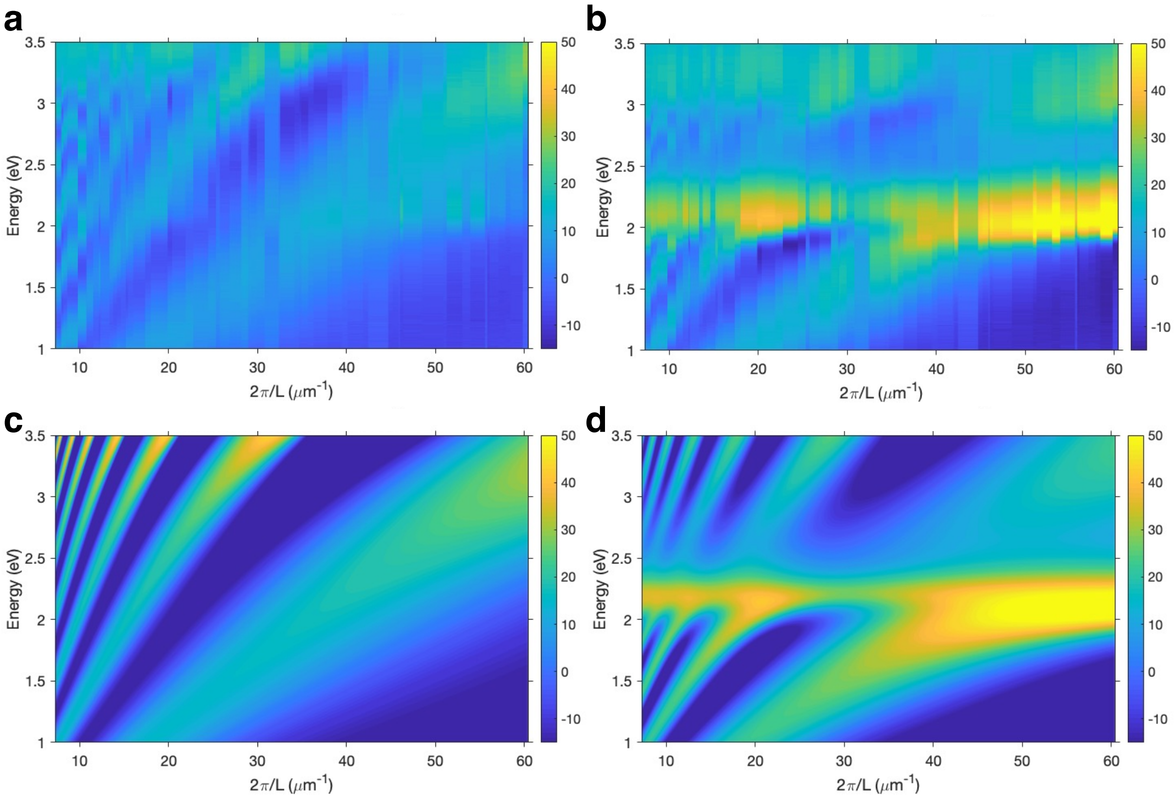

Figure S10: The ellipsometric parameter Δ plotted for SPI (a,c) and MC (b,d) films of thickness 104 nm $< L <$ 870 nm on an amorphous glass substrate ($\epsilon = 2.3$). The corresponding amplitude data is plotted in Figure S9. There is strong agreement between experiment (a-b) and modelling (c-d).

S10. Field profiles of Fabry-Perot and leaky modes

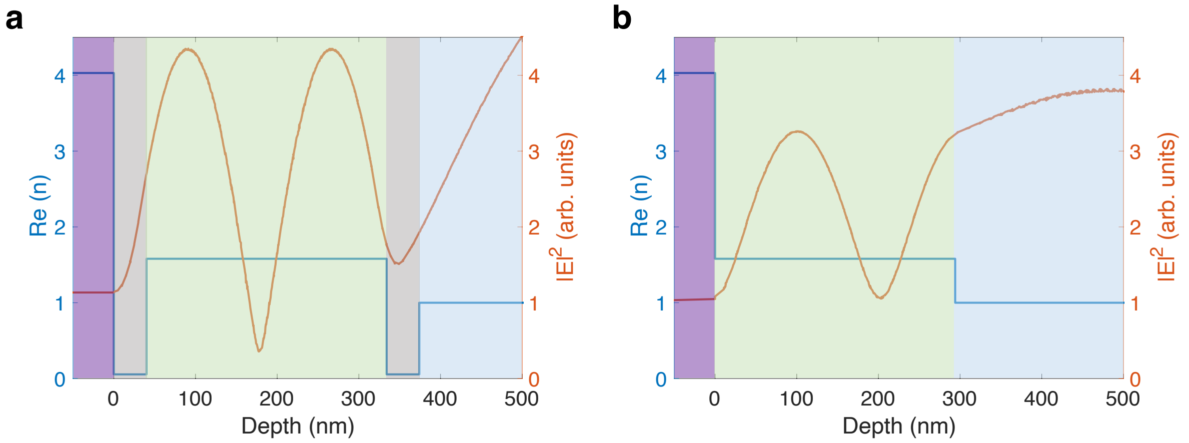

Figure S11: Electric field profiles associated with the second-order (a) Fabry-Perot cavity resonance in a Ag(40 nm)/SPI(294 nm)/Ag(40 nm) and (b) TE leaky mode in a SPI (294 nm) film, both on a Si substrate for light incident at $\theta = 65°$. The different coloured regions indicate each layer of the structure (Si, purple; SPI, green; air, blue; Ag, grey).

References

[1] Baum, W. A.; Dunkelman, L. Ultraviolet radiation of the high pressure Xenon arc. *J. Opt. Soc. Am.* **1950**, *40*, 782–786..

[2] CompleteEASE® 6.51. www.jawoollam.com/ellipsometry-software/completeease (J.A. Woollam Co., Inc.)

[3] Canales, A.; Baranov, D. G.; Antosiewicz, T. J.; Shegai, T. Abundance of cavity-free polaritonic states in resonant materials and nanostructures. *J. Chem. Phys.* **2021**, *154*, 024701, DOI: 10.1063/5.0033352

[4] Pockrand, I.; Swalen, J. D.; Santo, R.; Brillante, A.; Philpott, M. R. Optical properties of organic dye monolayers by surface plasmon spectroscopy. *J. Chem. Phys.* **1978**, **69**, 4001–4011.

[5] Pockrand, I.; Swalen, J. D. Anomalous dispersion of surface plasma oscillations. *JOSA* **1978**, *68*, 1147–1151.

[6] Pockrand, I.; Brillante, A.; Möbius, D. Exciton–surface plasmon coupling: An experimental investigation. *J. Chem. Phys.* **1982**, *77*, 6289–6295.

[7] Törmä, P.; Barnes, W. L. Strong coupling between surface plasmon polaritons and emitters: a review. **2015**, *78*, 013901.

[8] Kockum, A. F., Miranowicz, A., De Liberato, S., Savasta, S. & Nori, F. Ultrastrong coupling between light and matter. *Nat. Rev. Phys.* **2019**, *1*, 19–40.

[9] Thomas, P. A.; Tan, W. J.; Fernandez, H. A.; Barnes, W. L. A New Signature for Strong Light–Matter Coupling Using Spectroscopic Ellipsometry. *Nano Lett.* **2020**, *20*, 6412–6419.

[10] Azzam, R. M. A.; Bashara, N. M. *Ellipsometry and polarised light* North-Holland (Amsterdam), 1977.

[11] Menghrajani, K. S.; Barnes, W. L. Strong Coupling beyond the Light-Line. *ACS Photon.* **2020**, *7*, 2448–2459.

[12] Lawrence, C. R.; Sambles, J. R. Critical edge studies of highly absorbing Langmuir-Blodgett films. *Thin Solid Films* **1992**, *210-211*, 693-695.

[13] Barnes, W. L.; Sambles, J. R. Re-radiation from surface-plasmon-polaritons by surface roughness. *Solid State Commun.* **1985**, *55*, 921–923.

[14] Zengin, G.; Gschneidtner, T.; Verre, R.; Shao, L.; Antosiewicz, T. J.; Moth-Poulsen, K.; Käll, M.; Shegai, T. Evaluating Conditions for Strong Coupling between Nanoparticle Plasmons and Organic Dyes Using Scattering and Absorption Spectroscopy. *J. Phys. Chem. C* **2016**, *120*, 20588–20596.
